# Stereoscopic video deblurring transformer

**DOI:** 10.1038/s41598-024-63860-9

**Published:** 2024-06-21

**Authors:** Hassan Imani, Md Baharul Islam, Masum Shah Junayed, Md Atiqur Rahman Ahad

**Affiliations:** 1 Faculty of Engineering and Natural Sciences, Bahcesehir University, 34353 Istanbul, Turkey; 2 Department of Computing and Software Engineering, Florida Gulf Coast University, Fort Myers, FL 33965 USA; 3 Department of Computer Science and Engineering, University of Connecticut, Storrs, CT 06269 USA; 4 Department of Computer Science and Digital Technologies, University of East London, London, UK

**Keywords:** Mathematics and computing, Computer science

## Abstract

Stereoscopic cameras, such as those in mobile phones and various recent intelligent systems, are becoming increasingly common. Multiple variables can impact the stereo video quality, e.g., blur distortion due to camera/object movement. Monocular image/video deblurring is a mature research field, while there is limited research on stereoscopic content deblurring. This paper introduces a new Transformer-based stereo video deblurring framework with two crucial new parts: a self-attention layer and a feed-forward layer that realizes and aligns the correlation among various video frames. The traditional fully connected (FC) self-attention layer fails to utilize data locality effectively, as it depends on linear layers for calculating attention maps The Vision Transformer, on the other hand, also has this limitation, as it takes image patches as inputs to model global spatial information. 3D convolutional neural networks (3D CNNs) process successive frames to correct motion blur in the stereo video. Besides, our method uses other stereo-viewpoint information to assist deblurring. The parallax attention module (PAM) is significantly improved to combine the stereo and cross-view information for more deblurring. An extensive ablation study validates that our method efficiently deblurs the stereo videos based on the experiments on two publicly available stereo video datasets. Experimental results of our approach demonstrate state-of-the-art performance compared to the image and video deblurring techniques by a large margin.

## Introduction

Video deblurring is the process of restoring acute frames out of a blurry video. Deblurring is a crucial foundation for many computer vision tasks, and has therefore attracted significant research interest. Camera shake and object movement are common blur artifacts in dynamic video scenes^1,2^. In video processing, movement is critical, which causes most of the blur in a video, known as motion blur. Most approaches in this field first compute the motion between successive frames before applying frame transformations^3,4^. Consequently, the efficiency of the motion estimation profoundly influences the whole method’s functionality. Precise motion prediction, on the other hand, is complex and time-consuming^5^. Furthermore, most motion estimation algorithms address an optimization issue, slowing motion estimation. Some approaches use generative networks for video deblurring. For instance, Fanous et al.^6^ employed a generative adversarial network (GAN) for frame deblurring.

Limited research is reported in the literature for stereo video deblurring. In a recursive architecture, Pan et al.^7^ used stereo view information that a coarser depth or scene flow is used to calculate blur kernels. Some other studies employed stereo disparity and video motion. They estimated the disparity using data from the stereoviews and suggested a region tree technique for calculating the point spread functions (PSFs). Sellent et al.^8^ mention scene flow and stereo video deblurring as typical issues. Local homographs were employed to produce blur kernels using scene flow calculations, and scene flow and deblurring were addressed separately using pre-estimated scene flow.

Stereo video deblurring requires to preserve both disparity and temporal coherence. This makes it different from applying regular deblurring methods used for single images or standard videos. As a result, the motion information within successive frames potentially plays a considerable part in deblurring the frames next to them. Therefore, stereo video deblurring work can be divided into two significant components: (a) modeling symmetry cues across two viewpoints and (b) simulating sequences among subsequent frames. The intrinsic relation across pairs of stereo frames is exploited for modeling symmetry. Two considerations lead to our desire to propose a novel methodology for stereo video deblurring. Firstly, utilizing the motion information across succeeding frames and combining the information from adjacent frames of one perspective can aid in detecting distortions in pixels of the center frame. In fact, due to the slight movement between the few subsequent frames, surrounding frames can assist in deblurring the desired frame when deblurring a single video frame. Secondly, stereo vision provides two views simultaneously. Using the depth map, the equivalent pixels in one viewpoint can aid in the removal of blur in the comparable stereo view.

The transformer^1^ is well-known because of its capabilities in parallelization and outstanding modeling ability of the interconnections between the input sequences. It can potentially handle stereo video enhancement as a sequence modeling task^9^. Transformer-based approaches, such as Vision Transformers (ViT)^10^, break a video sequence into tiny areas and derive global connections among the token embeddings that reflect the areas. At the same time, spatial information is not granted considerable weight^2^. Such frameworks can only be used in a way that allows for stereo video deblurring, relying on local and texture information. Moreover, the ViT is not designed to resolve temporal dependencies and consistency, which are critical in the stereo-deblurring challenge.

To deal with motion blur, this study provides a novel Transformer-based stereo video deblurring approach that leverages nearby frames and information from the other corresponding stereo frame. Our Transformer-based stereo video deblurring approach leverages nearby frames and information from corresponding stereo frames to handle temporal information. We design an optical flow-based feed-forward layer to discover correlations across different video frames and align the features. Our approach employs a combination of spatial and temporal attention mechanisms to capture both local and global dependencies across frames. Specifically, we utilize a self-attention mechanism within each frame to model relationships between pixels, addressing spatial attention. Additionally, we introduce an optical flow-based feed-forward layer as a temporal attention mechanism to model relationships between consecutive frames, aiding the model in understanding the dynamics of the video sequence. By combining these two attention mechanisms, our architecture effectively captures both spatial and temporal dependencies in videos. We first estimate the motion information between consecutive frames using PWC-Net^11^ model. Then, after applying a 3D convolution, we perform a Transformer network to both stereo views. Then, the extracted features are fed to a CNN-based unit, and the features from the stereo frames are fused using a modified Parallax Attention Mechanism (mPAM) module. Lastly, a reconstruction layer creates the deblurred targeted frames. Due to the usage of both inter-view and intra-view frames, the temporal information of the video are handled in our method. The primary contributions to this paper are given below:

- We propose a new transformer model for deblurring stereoscopic videos. To deblur a target frame, the presented model incorporates the cross-view information and the information from nearby frames.
- In the model, we present a new feed-forward layer that spatially aligns features by calculating the relationships among all neighboring frames.
- We significantly improved the PAM module, namely mPAM, for combining features from stereo views to merge the stereo video features.
- Several image- and video-based deblurring methods are reimplemented to have a fair comparison with the proposed method based on two benchmark datasets. Experimental results and ablation studies show the superiority of our method compared to the previous art.

In Section “Related works”, we briefly illustrate essential methods related to 2D and 3D images and video deblurring. We describe the proposed model and its different parts in Section “Proposed method”. Section “Datasets and experiments” discusses the experimental setup, implementation, and datasets. The efficiency of our method is evaluated in Section “Results and discussions”. Finally, we conclude the paper with some future work guidelines.

## Related works

This section briefly discusses the relevant single, stereo image, and video deblurring methods.

### 2D image deblurring

Certain classic methods for removing the blur from a single image are proposed and available in the literature. Some examples include the L0 regularized prior^12^, the dark channel prior^13^, and the discriminative prior^14^. These methods have several limitations in representing spatial blur in dynamic settings. These methods often struggle to represent complex, spatially-varying blur in dynamic scenes with motion. However, several methods, including^15–17^, used the depth map to simulate the blur distortion that is not homogeneous. Because of the time-consuming optimization process, such methods are expensive.

Traditional deblurring methods are computationally expensive^18^. For dealing with commonly occurring blur resulting from the relative movement of the object-camera, Nah et al.^19^ developed a no-reference solution. This method is a CNN-based multi-scale system that attempts to recover frames with more details. The approach suggested in^20^ involves gradually recovering the image at various qualities from providing a strategy that is less complicated than earlier techniques and performs better. A multi-scale structure has been included in the suggested paradigm. Zhang et al.^21^ presented a strategy for dealing with the spatially variable blur, which occurs as the camera moves. Three CNNs and one RNN were employed. Liang et al.^22^ approached the deblurring problem from another perspective. They proposed a new model for deblurring raw images. They also used a new raw image deblurring dataset and trained their model on that dataset. In another study, Honorvar et al.^23^ proposed a new model of PSF of motion blur to analyse the motion invariant in frequency and moment domains.

If the blur is not uniformly distributed, for example^24,25^, employed CNNs to predict the blurry regions. In^26^, the authors developed a new approach for detecting motion blur caused by camera and object movements. They designed a new multi-scale CNN-based framework with certain skip connections to manage data generation. Recently, an Edge-Aware Scale-Recurrent Network (EASRN) was presented by Chang et al.^27^ to deal with the motion blur in the presence of the outliers that deblurred the frames at different scales. This method also trained a deep model to restore the high-quality edges. Li et al.^28^ developed a CNN-based model for image deblurring based on depth information estimation. Then, they use a feature transform model to extract depth features and combine them with spatial features. It demonstrated that depth information could be effectively utilized for image deblurring. Very recently, Restformer^29^ is proposed which is a Transformer-based model. They design multi-head attention and feed-forward blocks to capture long-range pixel interactions. In another recent study, Kong et al.^30^ introduced a frequency domain-based Transformer for deblurring images. Instead of matrix multiplication, they calculate the scaled dot-product attention using their proposed product method.

### 2D video deblurring

Several recent works have addressed 2D video deblurring^31–36^. Delbracio et al.^31^ used the Fourier transform to fuse the data from neighboring frames in a video to remove motion blur. The neighboring frames are registered for each frame, and then the registered frames are combined using the Fourier transform. CNN’s are one of the most successful methods developed for video deblurring. For example, an encoder-decoder-based model is applied to the batch of neighboring frames for deblurring in^32^. The method in^33^ proposed a Spatio-temporal 3D CNN model to deblur videos. Zhang et al.^36^ modeled the temporal dependencies using a non-local layer that calculated the similarities and differences between frames with a recursive block.

Pan et al.^34^ proposed an optical flow-based model in another study. This method learns CNN to calculate the optical flow and reconstructs the deblurred frames afterward. Son et al.^35^ are also based on using neighboring frames. They proposed a novel motion estimation method that is invariant to blur. Instead of warping frames for compensating motion, they used a pixel volume to to use the most sensitive pixels of the blurred video. Recently, Wang et al.^37^ presented a CNN-based model, providing spatial-temporal and frame channel attention modules and a reconstruction block to re-create the high-resolution frames. Video deblurring and optical flow (VDFlow)^38^ estimated optical flow and deblurring at the same time. This model has two parts: encoder-decoder for deblurring and optical flow network (FlowNet)^39^ for optical flow estimation. In another study, Chen et al.^40^ formulated deblurring as a residual learning problem. They trained a U-net model to deblur the frames and then iteratively generated frames to create a high frame-rate video.

### Stereo image and video deblurring

Some studies have employed disparity and motion (for video) to deblur stereo content. The depth information and point spread functions were calculated in^41^. They estimated the depth of information and then suggested a region tree approach for computing the point spread functions^8^ used scene flow estimates to generate blur kernels and a grading approach to the borders of moving objects. In contrast, Pan et al.^7^ combined scene flow estimation with deblurring and discovered that motion and blur distortions could interact. Network with depth awareness and view aggregation (DAVANet)^42^ was proposed for stereo image deblurring. It includes three major sections: an encoder-decoder backbone, a disparity prediction model, and an integration framework that combines the two networks to generate deblurred frames. They also presented the Stereo Blur dataset. Recently, UNet-Deblur^43^ introduced a CNN-based stereo video deblurring approach that considered the stereo frames in succession. They fed the target and successive neighboring frames to the 3D CNN model to adjust for motion in stereoscopic video, which can aid with more deblurring. After compensating for motion across subsequent frames, the left and right frames are subjected to a 3D CNN to extract their features. They redesigned 3D U-Nets to use them as feature extractors. The PAM^44^ module is adjusted to fuse cross-view information and construct the output deblurred frames to combine the left and right information. Besides, despite having deeper architecture compared to the other stereo image-based methods such as DAVANet^42^, their method has poor efficiency. Motivated by this, we develop a new architecture to better utilize the neighboring and stereo information to deblur the stereo video frames efficiently.

**Figure 1:**
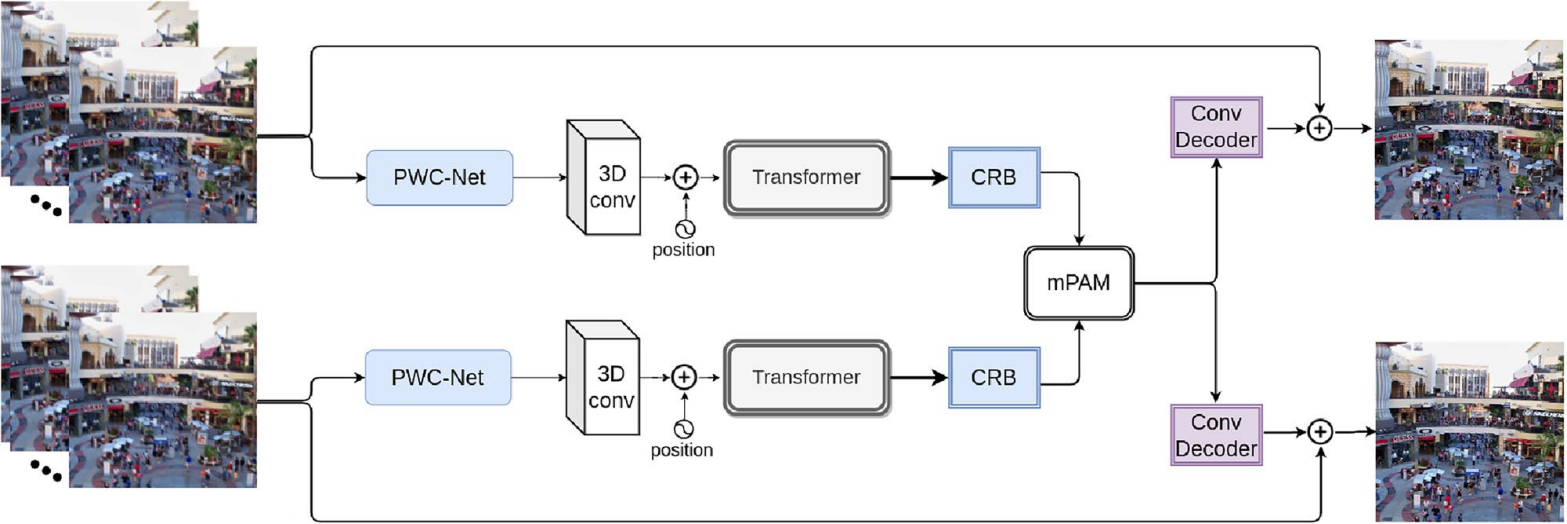
The proposed stereo video deblurring model. Firstly, PWC-Net estimates the motion between the neighboring frames. Then, we apply a 3D CNN layer to the motion-compensated frames, and the proposed Transformer model accepts the resulting features as input. Next, another CNN layer (CRB) extracts deep features. The mPAM then fuses the stereo input features. A convolutional decoder constructs the deblurred frames from the left and right features. Finally, we form the output by adding the blurry middle target frames with the reconstructed left and right frames.

## Proposed method

Figure 1 shows the design architecture of our stereo video deblurring approach. We estimate the motion between succeeding center frames using the pyramid, warping, and cost volume network (PWC-Net)^11^. After warping the neighboring frames to the center frames, we apply them into a 3D convolutional block, which extracts even more localized characteristics. A Transformer network then learns the features from the middle and motion-compensated frames. We use four convolutional residual blocks (CRB) to extract more deep features. The CRB provides features with broad receptive fields and intense sampling rates, which help to estimate stereoscopic matching. Then, we combine the cross-view features with modifying the PAM^44^. Finally, a batch of 2D convolutional blocks reconstructs the target frames and further adds the middle frames. We first discuss PWC-Net Architecture, and then we discuss the proposed Transformer Architecture.

### PWC-Net and transformer architecture

#### PWC-Net Architecture

**Figure 2:**
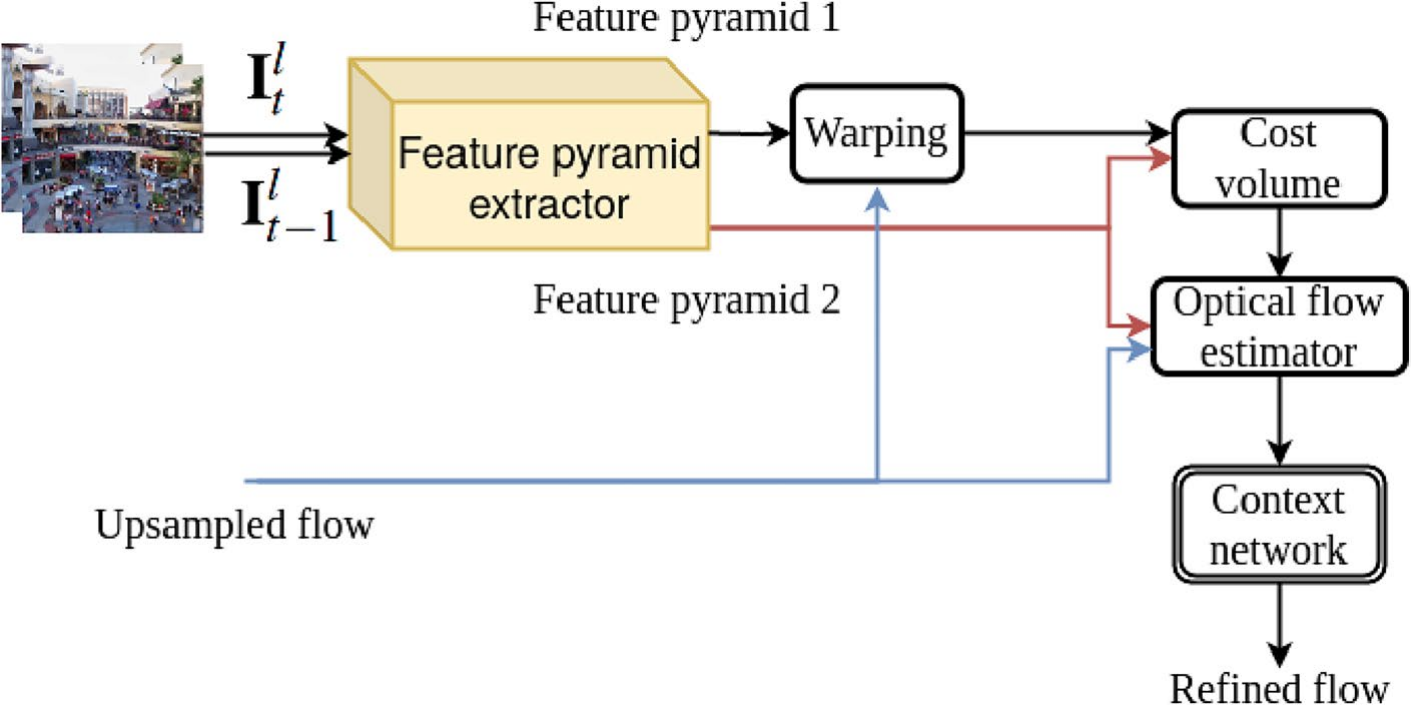
Feature pyramid in PWC-Net^11^. The arrows represent the flow estimation direction, while the pyramids are built in reverse directions. PWC-Net uses the upsampled flow to warp features in the neighboring frame, calculates a cost volume, and processes it with neural networks.

We utilize PWC-Net, which is built upon fundamental principles: pyramidal processing, warping, and leveraging a cost volume. Implemented within a trainable feature pyramid, PWC-Net utilizes the existing optical flow estimation to deform the CNN features of the subsequent image. It then combines these deformed features with those from the initial image to create a cost volume. This volume is then analyzed by a CNN to estimate the optical flow. Optical flow approximation is fundamental in vision tasks with several use cases^45^. The energy reduction strategy proposed by Horn and Schunck^46^ is used by state-of-the-art approaches. Nevertheless, optimizing a complicated energy function is typically costly for real-world use cases. Figure 2 summarizes the major parts of PWC-Net. First, we calculate the feature pyramids to extract features at different scales. Let (${\textbf {I}}_{t}^{l}$ and ${\textbf {I}}_t^{r}$) and (${\textbf {I}}_{t-1}^{l}$ and ${\textbf {I}}_{t-1}^{r}$) represent the two stereo consecutive frames. Pyramid extraction includes six levels, with 16, 32, 64, 96, 128,  and 196 number of features^11^. The calculated pyramids are as follows: ${\textbf {P}}_{t}^{l}$, l=0,...,5. Then, another layer performs the warping process. For stereo frames, the features of ${\textbf {I}}_{t}^{l}$ and ${\textbf {I}}_t^{r}$ are warped using the features of ${\textbf {I}}_{t-1}^{l}$ and ${\textbf {I}}_{t-1}^{r}$, and the up-sampled flow of the upper pyramid level from the l+1th level for each view:

1 $$\begin{aligned} \begin{array}{l} {{{P}_t^{l}(i) = {P}_{t-1}^{l}(i + up(t^{l+1})(i))}} \\ \end{array} \end{aligned}$$

In this equation, *i* and *up* are the pixel index and the upsample operators, respectively. Here, the bilinear interpolation calculates the warps.

**Figure 3:**
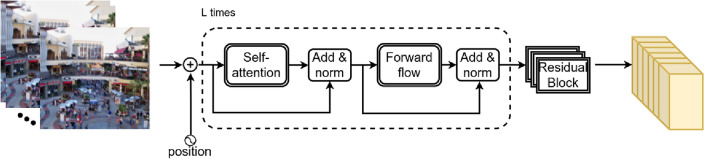
The Transformer’s high-level design structure. To extract information from the frames, we use convolutional layers. The self-attention and feed-forward optical flows are applied after position encoding, utilizing the add and normalization blocks. Finally, residual modules create the desired outputs.

Figure 3 depicts the Transformer’s high-level architecture. Firstly, we apply a 3D CNN to the stereo batches to transfer the input frames (${\textbf {I}}_3^{l_{comp}}$ and ${\textbf {I}}_3^{r_{comp}}$) from 3 to 64 output channel (${\textbf {I}}_{64}^{l_{comp}}$ and ${\textbf {I}}_{64}^{r_{comp}}$). Next, we calculate the initial features using residual modules (${\textbf {I}}_{64}^{l_{Res}}$ and ${\textbf {I}}_{64}^{r_{Res}}$). The added and normalized blocks connect attention and flow with residual layers. As seen in Fig. 3, we repeat these layers *L* times and apply another residual block. We discuss the transformer’s sub-blocks in the following sub-sections.

**Figure 4:**
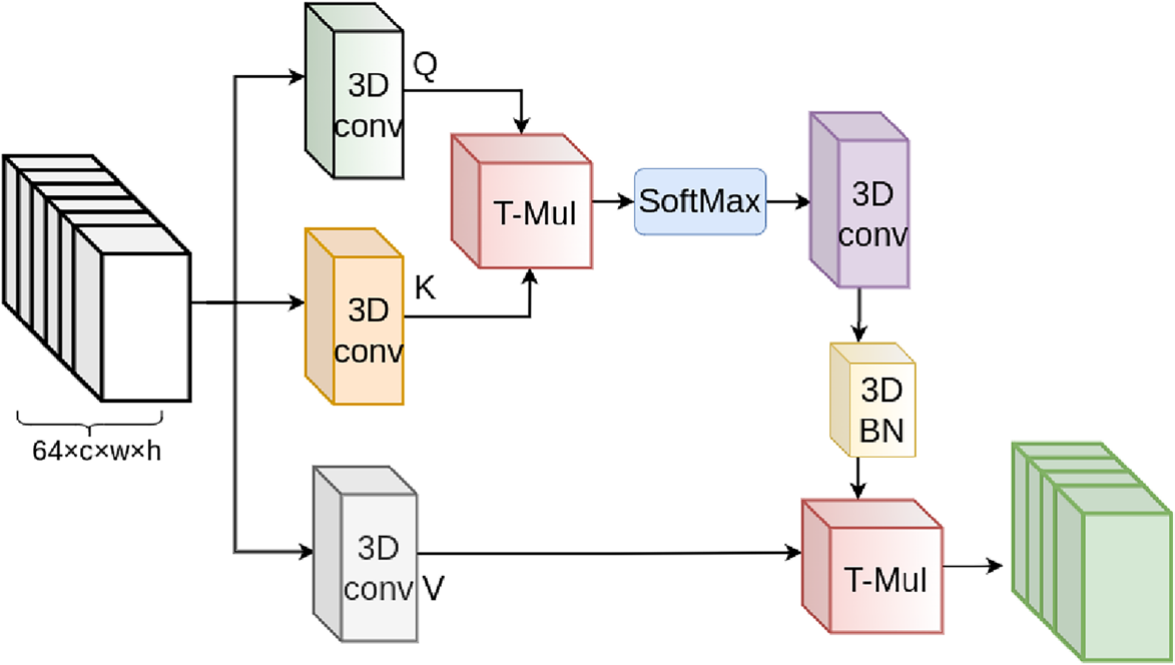
The self-attention module’s architecture. The input features from a 3D CNN module build the tensors *Q*, *K*, and *V*, and after tensor multiplications, we create the output.

**Figure 5:**
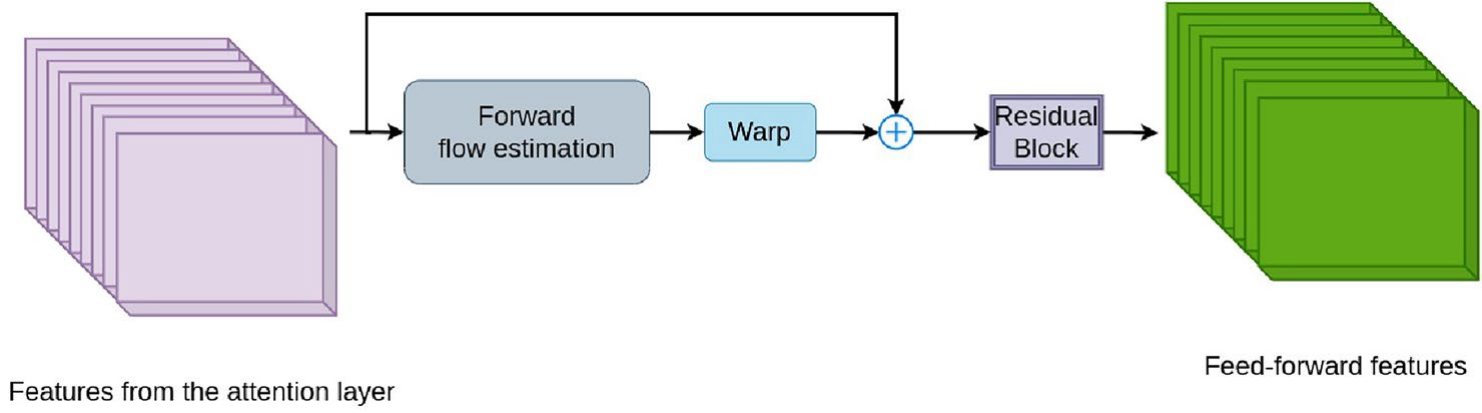
Architecture of the optical flow-based feed-forward layer: Firstly, the features coming from the self-attention layer estimate the forward optical flows. Then, after the warping operation, residual and convolutional layers create the output features.

#### Self-attention layer

Figure 4 depicts the architecture of this layer. We start with creating the Query (*Q*), Key (*K*), and Value (*V*) tensors. With applying a 3D CNNs to (${\textbf {I}}_{64}^{l_{Res}}$ and ${\textbf {I}}_{64}^{r_{Res}}$), we generate *Q* (${Q}_{64}^{l}$ and ${Q}_{64}^{r}$) and K tensors (${K}_{64}^{l}$ and ${K}_{64}^{r}$) to extract their feature maps. 64 filters with size of 3$\times$3$\times$3 and padding of 1 perform to 3 CNNs. Therefore, *Q*, *K*, and *V* for the left channel are as follows:

2 $$\begin{aligned} \begin{array}{l} Q = 3D{CNN}(K_1,{\textbf {I}}_{64}^{l_{Res}})\\ K = 3D{CNN}(K_2,{\textbf {I}}_{64}^{l_{Res}})\\ V = 3D{CNN}(K_3,{\textbf {I}}_{64}^{l_{Res}})\\ \end{array} \end{aligned}$$

where ${K}_{1,2,3}$ are CNN kernels. Next, we calculate the similarity tensor using the tensor product (*TP*) for the left video:

3 $$\begin{aligned} \begin{array}{l} QK_l = SM(\textit{TP}(Q^T,K))\\ \end{array} \end{aligned}$$

where SM is the softmax operation. We apply the output features into a 3D CNN including 64 filters and 3$\times$3$\times$3 kernel size. Next, we multiply the results by *V* and combine them with the input features to obtain the attention layer’s output features for the left video:

4 $$\begin{aligned} \begin{array}{l} {Attn_l = {\textbf {I}}_{64}^{l_{Res}} + \textit{TP}(QK_l,V_l)}\\ \end{array} \end{aligned}$$

The calculations for the right features are identical to the left one.

#### Position encoding

The permutation is unchanging in the original Transformer architecture^47^, but in deblurring task, the position is crucial. In this paper, we use the positional encoding in^48^. For left and right Transformers, we utilize *d*/3 sine and cosine with distinct frequencies for each spatial coordinate:

5 $$\begin{aligned} \begin{array}{l} {{PE}_{l}(pos_l,i) = \left\{ \begin{array}{ll} sin(pos_l . w_k) &{} \quad \text {for}\quad {i=2k}, \\ cos(pos_l . w_k) &{} \quad \text {for}\quad {i=2k+1}; \end{array} \right. } \end{array} \end{aligned}$$

where ${pos}_{l}$ is the position in the dimension for the left Transformer, and ${w}_{k} = 1/10000^{2k/({d/3})}$^48^.

#### Feed-Forward (FF) Layer

The fully connected FF does not utilize the interdependence across tokens of neighboring frames. We propose an optical flow-based approach to align the input features in the spatial dimension, considering the relations between successive frames. Figure 5 describes the proposed architecture. We apply the feature maps from ${Attn}_{l}$ and ${Attn}_{r}$ to this block. We use spatial pyramid network (SpyNet)^49^ to estimate the motions across frames *n* and *m* as ${flow}_{l}$ and ${flow}_{r}$:

6 $$\begin{aligned} \begin{array}{l} {flow}_{l}(m,n) = \left\{ \begin{array}{ll} \left[ 0\right] _{W \times H} &{} \quad \text{for}\quad {m=n}, \\ spy({\textbf {I}}_n^{l}, {\textbf {I}}_m^{l}) &{} \quad \text {for}\quad {m\not =n}; \end{array} \right. \\ {flow}_{r}(m,n) = \left\{ \begin{array}{ll} \left[ 0\right] _{W \times H} &{} \quad \text{for}\quad \textit{m=n}, \\ spy({\textbf {I}}_n^{r}, {\textbf {I}}_m^{r}) &{} \quad \text{for}\quad m \not =n; \end{array} \right. \end{array} \end{aligned}$$

where *spy* is the SpyNet^49^, and *LR* Next, we warp the features in the forward direction:

7 $$\begin{aligned} \begin{array}{l} FF_l = warp({Attn}_{l},{flow}_{l}) \\ FF_r = warp({Attn}_{r},{flow}_{r}) \end{array} \end{aligned}$$

Next, we combine the ${FF}_l$ and ${FF}_r$ with ${Attn}_{l}$ and ${Attn}_{r}$. To build the connection between succeeding frames, we suggest using a CNN-based forward layer. To construct the resulting features of this module, we particularly employ residual blocks with a 3D CNN at the end. The following is how we define a fully connected feed-forward layer:

8 $$\begin{aligned} \begin{array}{l} FF_l^{o}({Attn}_{l}) = conv(LN( {Attn}_{l} + Res([{Attn}_{l},FF_l]))) \\ FF_r^{o}({Attn}_{r}) = conv(LN( {Attn}_{r} + Res([{Attn}_{r},FF_r]))) \\ \end{array} \end{aligned}$$

**Figure 6:**
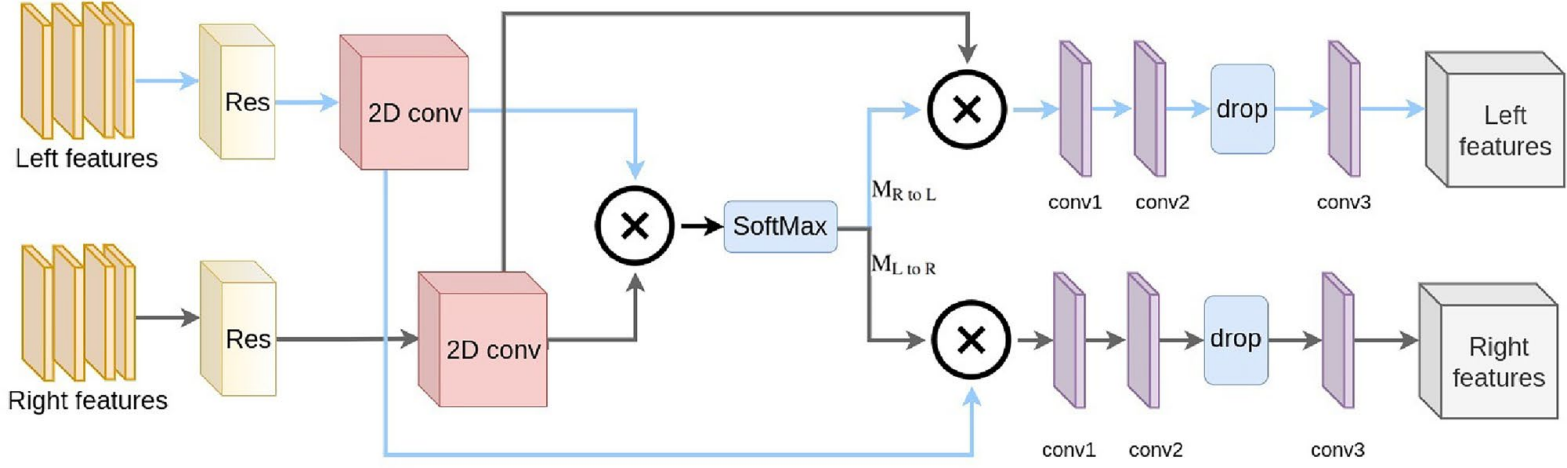
The mPAM flow diagram: Firstly, the stereo input features are input to the residual layer (Res). After applying a 2D CNN, we fuse the cross-view information and create the output.

### Modified PAM (mPAM)

Stereo video frame pairs offer an opportunity to enhance the effectiveness of image and video deblurring by providing supplementary information from a second perspective. Nonetheless, integrating this data presents challenges due to the considerable variations in disparities between stereo images. To address this, we propose a parallax-attention mechanism (PAM) featuring a global receptive field along the epipolar line. This mechanism aims to manage diverse stereo video frames with substantial differences in disparity effectively. Parallax Attention Mechanism (PAM)^44^ merges the features of stereo images. We improve the PAM design to account for the input 3D features representing video sequences over time. The input features to the mPAM module are 3 dimensional (from left or right videos). Therefore, 3d residual features at first, then apply 2D convolutions. As shown in Fig. 6, the left and right features are fed to the 3D residual blocks (Res). 2D convolutions (2D conv) are applied next to make the input suitable for 3D features. Tensor multiplication is then performed to the left and right features. SoftMax block then creates the attention maps: M_R to L_ (from right to left) and M_L to R_ (from left to right). Next, for all disparities, we combine the summation of features with the former right features. We removed valid mask generation from PAM structure in^44^, because the authors use an occlusion detection method to generate valid masks. Since this operation adds to the computations, we removed it from the main algorithm. To generate deeper features suitable for deblurring, we utilize 3 CNN layers. There are 128 filters in the initial 2D convolution *conv1*. For this convolution, we employed a 5$\times$5 kernel size. Just by changing the kernel size to 3$\times$3, the *conv2* is similar to the *conv1*. Then, at a rate of 0.5, we apply a dropout layer *drop*. The third layer *conv3* is with 64 filters and a 3$\times$3 kernel.

### Loss functions

Five loss functions are defined in this section which we use for model training. The mean absolute error (MAE) is the first loss, which determines the differences among the original and deblurred frames. The average MAE of the stereo viewpoints is as follows:

9 $$\begin{aligned} mae_{loss} = (mae_l + mae_r)/2 \end{aligned}$$

In addition, we exploit photometric ($p_{loss}$) and cycle ($c_{loss}$) losses^44^. To consider the smoothness in correspondence space, we use smoothness loss as follows:

10 $$\begin{aligned} \begin{aligned} s_{loss}&= \sum _{A} \sum _{i,j,k} (|| A(i,j,k) - A(i+1,j,k) ||_1 \\&\quad + || A(i,j,k) - A(i,j+1,k+1) ||_1) \end{aligned} \end{aligned}$$

where, *A* is the cross-view attention maps. Finally, stereo consistency loss $sConsist_{loss}$ considers the stereo consistency between deblurred stereo frames. For stereo consistency, we calculate the end-point error (EPE) using Euclidean distance among the two disparities of the original and deblurred video frames. The resulting loss is as the union of defined five losses:

11 $$\begin{aligned} \begin{aligned} loss&= mae_{loss} + \gamma (p_{loss} + c_{loss} + s_{loss} \\&\quad + sConsist_{loss}) \end{aligned} \end{aligned}$$

where, $\gamma$ is a constant which is set as 0.05.

## Datasets and experiments

To train the proposed deblurring model, we utilize the only publicly available dataset of the Stereo Blur dataset^42^. For model evaluation, we use the test set of Stereo Blur and LFOVIAS3DPh2^50^ datasets that are discussed in the following subsections.

### Datasets and evaluation criteria

#### Stereo blur ^42^ dataset

This dataset contains videos of objects and people with minor disparities. The outdoor videos include humans, cars, boats, and outdoor scenarios. Furthermore, the dataset contains videos captured in various situations, such as lighting and weather variations. The authors expanded the dataset to include a variety of motion settings utilizing three distinct imaging styles: handheld, stationary, and onboard shots. The ZED stereo camera^51^ is being used to create this dataset, with an FPS of 60. The stereoscopic video has identical arrangements on both stereo sides. It includes masks for eliminating faulty samples in the disparity and distorted frame segments, generated using the bidirectional consistency check^52^. In this dataset, there are 135 stereo videos.

#### LFOVIAS3DPh2 ^50^ Dataset

It is used for stereoscopic video quality assessment^53–55^ and contains 12 pure and 288 distorted videos. These videos were recorded with a Panasonic camera, and their resolution is $1920\times 1080$. High-quality videos are labeled with a high value and vice versa (ranging from 5 for the highest quality and 0 for the lowest grade). All the videos have an exact duration of 10 seconds. Since the LFOVIAS3DPh2 dataset contains blurry and original videos, we use this dataset’s blurry videos to evaluate our stereo video deblurring method. To make blurry videos, the authors in^50^ employed ffmpeg’s *box blur* function. They created 72 blurry stereo videos by applying 3 blur levels to the 12 reference stereo videos.

#### Evaluation metrics

We compare our model’s performance to deep learning-based and classical approaches in the two popular Structural SIMilarity Index (SSIM) and Peak Signal-to-Noise Ratio (PSNR) metrics.

### Experimental setup

To train the proposed model, we firstly center crop the left and right frames with 256 pixels and construct a dataset with a size of $256\times 256$. Our computing system’s configurations are NVIDIA RTX 3090 GPU, 24GB of GPU RAM, and i9-10850K CPU 3.60 GHz. We utilize the Adam optimizer^56^ with ${\beta }_{1}$=0.9 and ${\beta }_{2}$=0.99. We employ a batch size of 10 with the learning rate of 0.001, and we trained the model for 528k iterations.

## Results and discussions

To our best knowledge, only UNet-Deblur^43^ as a video-based stereo deblurring method reported results on the Stereo Blur dataset. As a result, we do comparisons with this method, stereo image-based approaches, and some video and image deblurring methods. In Zhou et al.^42^, the models of^19–21,57^ are trained on the Stereo Blur dataset. Tables 1 and 2 demonstrate the outcomes of the analysis of image- and video-based deblurring approaches for Stereo Blur and LFOVIAS3DPh2^50^ datasets, respectively.

**Table 1:** Comparison of our proposed method with image- and video-based deblurring methods in terms of PSNR, SSIM, and time-complexity on the Stereo Blur^42^ dataset.

Methods	PSNR	SSIM	Time (s)	Params (M)
Image-based Methods				
Whyte^58^	24.48	0.8410	700	–
Sun^24^	26.13	0.8830	1200	7.26
Gong^25^	26.51	0.8902	1500	10.29
Nah^19^	30.35	0.9294	4.78	11.71
Kupyn^57^	27.81	0.8895	0.22	11.38
Zhang^21^	30.46	0.9367	1.40	9.22
Tao^20^	31.65	0.9479	2.52	8.06
DAVANet^42^	33.19	0.9586	0.31/pair	8.68
2D Video-based Methods				
Pan et al.^34^	33.78	0.9572	0.42/pair	32.4
Son et al.^35^	33.22	0.9328	0.25/pair	21.02
Stereo Video-based Methods				
UNet-Deblur^43^	30.56	0.9221	0.57/pair	19.9
**Ours**	**34.06**	**0.9742**	0.81/pair	38.4

The best results are in bold. The “–” is used for unavailable information.

**Table 2:** Comparison of our proposed method with image- and video-based deblurring methods in terms of PSNR and SSIM on the LFOVIAS3DPh2^50^ dataset.

Methods	PSNR	SSIM
Image-based methods		
Kupyn^57^	27.12	0.8770
DAVANet^42^	32.1073	0.9394
2D video-based methods		
Pan et al.^34^	32.0331	0.9387
Son et al.^35^	31.9880	0.9355
Stereo video-based methods		
UNet-Deblur^43^	28.7216	0.9018
**Ours**	**32.2601**	**0.9410**

The best results are in bold.

### Quantitative results

We compare the proposed method’s effectiveness with the available 2D and 3D image and video-based methods in Table 1, notably the only available stereo video deblurring method^43^. The results demonstrate that our model improved by 3.50 dB in PSNR and 0.0521 dB in SSIM, which significantly improved. Furthermore, stereo video deblurring approaches of Sellent et al.^8^ and Pan et al.^7^ are not open-source, and their results on the Stereo Blur dataset have not been published. They conducted their research using videos that they created for their experiments. Sellent et al.^8^ created stereo images for their experiments, which is not possible to use in our experiments since our method requires some successive frames. Our algorithm requires at least 5 successive frames. In addition, it contains a few images, which means it cannot train our deep learning-based model. Since the training code for^7^ is not available, we could not compare our results with it. To facilitate comparison, we re-implemented two 2D video deblurring approaches of Son et al.^35^ and Pan et al.^34^. Pan et al.^34^ efficiently use domain knowledge of video deblurring. Still, our method outperforms this method thanks to using the mPAM module. Compared to Son et al.^35^ model, we improve 0.83 and 0.27 dB in PSNR on Stereo Blur and LFOVIAS3DPh2 datasets, respectively. DAVANet^42^ is a stereo image deblurring method that performs better than the other image-based methods by a large margin. We also compare PAM^44^ with the proposed mPAM inside our whole model. Table 5 compares the effects of these two modules on the effectiveness of the proposed stereo video deblurring method. The mPAM improves the PSNR by 0.59 dB.

**Figure 7:**
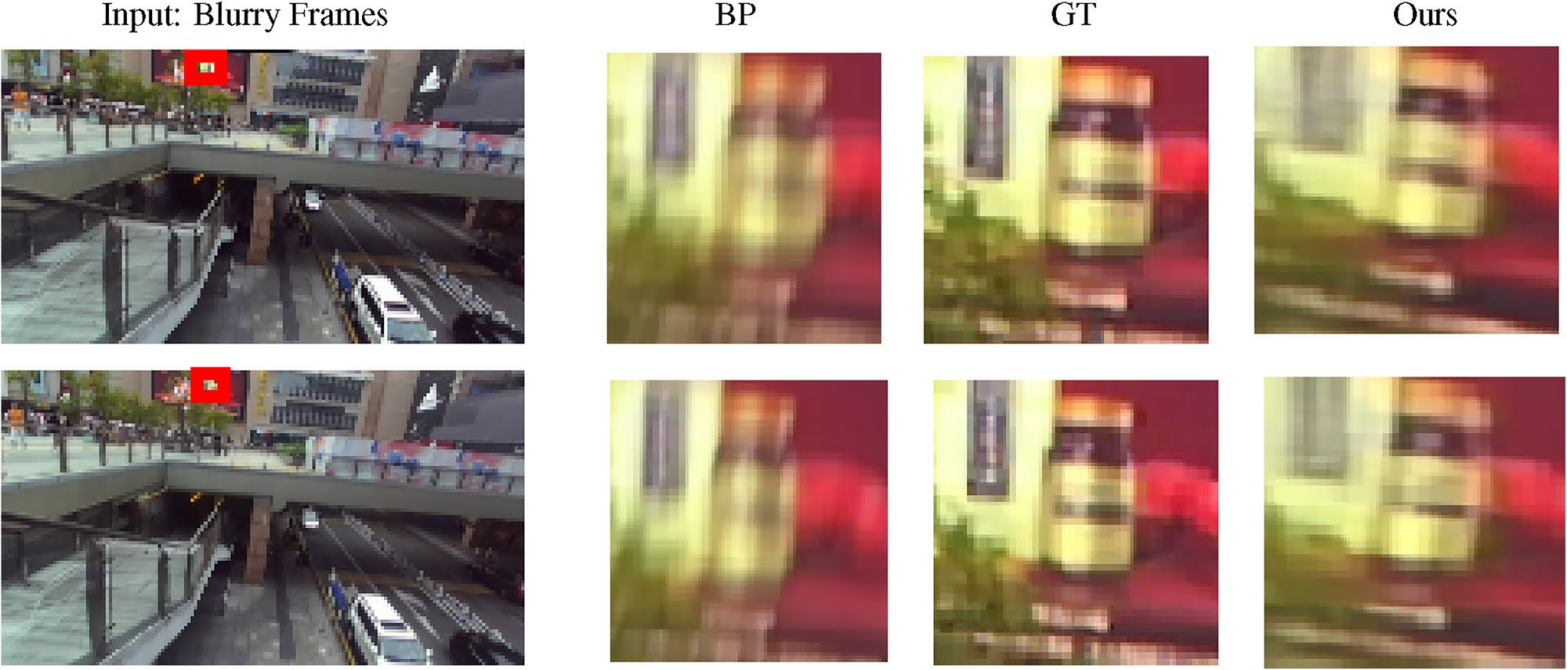
Qualitative performance of the proposed method on the Stereo Blur^42^ dataset. The first row displays the left frame, and the second row displays the right frame of a sample test video. The BP and GT refer to the selected Blurry Part (BP) and Ground Truth (GT) of the video frame. .

**Figure 8:**
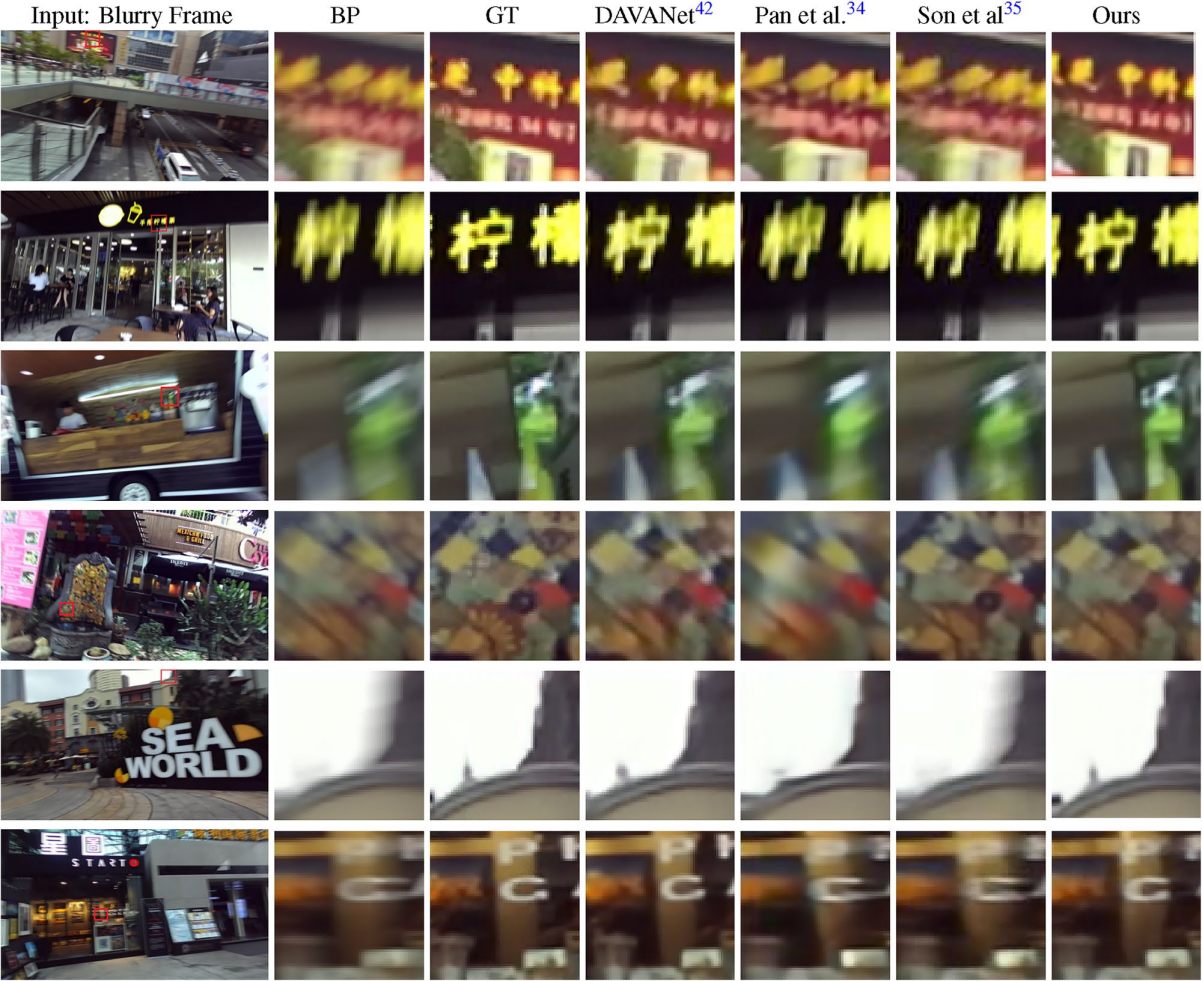
Qualitative performance comparison with state-of-the-art methods on different indoor and outdoor video frames in the Stereo Blur^42^ dataset. The BP and GT refer to the selected Blurry Part (BP) and Ground Truth (GT) of the video frames.

**Figure 9:**
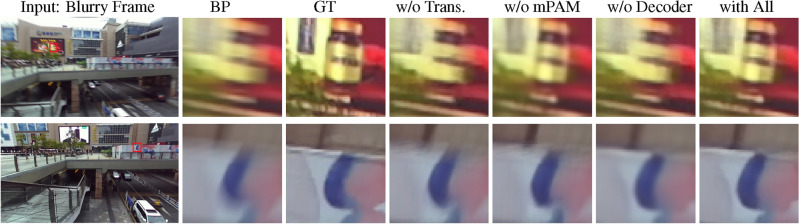
Qualitative performance comparison of our method, with and without different contributing modules, on two video frames on Stereo Blur^42^ dataset. BP and GT refer to the selected Blurry Part of the frame and Ground Truth frame, respectively.

**Table 3:** Stereo consistency.

Stereo-based methods	Params (M)	EPE
DAVANet^42^	33.19	0.7380
UNet-Deblur^43^	30.56	0.7584
Ours	34.06	0.7196

The average EPE of the proposed method against the stereo-based methods.

### Stereo consistency

To calculate the consistency between deblurred and original stereo video frames, we further investigate the end-point error (EPE) using the Euclidean distance among the two disparities (in original and deblurred videos), we estimate the disparity between the stereo frames of the reference videos with the approach proposed in Hirschmuller et al.^59^ before calculating the disparity of the deblurred video frames. We calculate the EPE between two disparity values as the Euclidean distance between them. The results are shown in Table 3. The average EPE of our method is 0.7196 on the Stereo Blur dataset. In comparison, DAVANet^42^ receives the average EPE of 0.7380 on the same dataset. Our method maintains better stereo consistency in the deblurring results.

**Figure 10:**
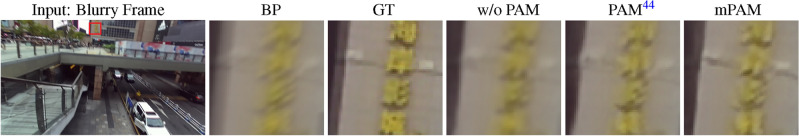
Effect of different PAM configurations in the overall performance of the proposed method on a video frame from Stereo Blur^42^ dataset: w/o PAM: without PAM in our model, PAM^44^, mPAM: modified PAM. The BP and GT refer to the selected Blurry Part (BP) and Ground Truth (GT) of the video frame.

### Qualitative results

Figure 8 demonstrates the qualitative performance of our method on some stereo video frames from the Stereo Blur dataset. We compare our results with two 2D video deblurring methods (Son et al.^35^, Pan et al.^34^), and one stereo image deblurring method, namely DAVANet^42^). We selected six video frames for this comparison, and in most of them, our method qualitatively outperforms the other methods. This figure shows that our approach efficiently uses the data from the neighboring frames. When the frame is blurry, the nearby frames help to deblur the middle frame. Additionally, Figure 7 illustrates the performance of the proposed method in stereo settings on the Stereo Blur dataset. The first row depicts the left frame, while the second row shows the right frame of a sample test video.

**Table 4:** Performance comparison with (w) and without (w/o) contributing modules on Stereo Blur^42^ dataset.

Model settings	Params (M)	PSNR	SSIM
w/o Trans.	21.4M	30.13	0.9359
w/o mPAM	36.4M	30.39	0.9378
w/o Decoder	16.7M	31.44	0.9461
With all modules	38.4M	**34.06**	**0.9742**

Significant values are in bold.

**Table 5:** Comparison the performance between the PAM^44^ and the mPAM on Stereo Blur^42^ dataset.

Model settings	Params (M)	PSNR	SSIM
PAM^44^	38.1M	33.47	0.9568
mPAM	38.4M	34.06	0.9742

### Ablation studies

We perform an extensive ablation study on the Stereo Blur^42^ dataset to analyze the impact of various components within our model. This involves systematically removing specific modules (i.e., Transformer, mPAM module, Decoder, and a consecutive number of frames) and evaluating the resulting effect on the model’s performance (PSNR and SSIM) as shown in Table 4 and Fig. 9. We refer to the architecture in Fig. 1 for this analysis.

#### Effect of the transformer

We remove the Transformer from both the left and right channels to see the effectiveness of our model performance. Since the left (PWC-Net) and right (CRB) sides of the Transformer in Fig. 1 contain 3D and 2D CNNs, respectively, we cannot directly remove the Transformer. Let’s say the output of the PWC-Net is a 5 dimensional tensor with size ($Batch-size, N-frames, N-channel, W, H$). We reshape the tensor to make the input tensor with 4 dimensions ($Batch-size,$ $N-frames \times N-channel$,*W*, *H*), then apply it to the CNN network. The results are shown in the first row of Table 4. The Transformer has a notable influence on the model efficiency, and the model PSNR decreases from 34.06 to 30.13 after removing the Transformer from the left and right channels. As shown in Fig. 9, when we disable the Transformer module, the model performance drops essentially.

#### Effect of the mPAM

We further investigate the effect of the cross-view information and deblur the left and right frames independently without considering the mPAM module. The model performs similarly to two models trained separately without using the cross-view information. The result of this change is illustrated in the second row of Table 4. Even without using the cross-view information, the proposed method outperforms image-based methods of Whyte^58^, Sun^24^, Gong^25^, Nah^19^, and Kupyn^57^. However, DAVANet^42^, which uses the cross-view information efficiently, performs better than the proposed method without the mPAM module. Our model effectively uses the cross-view information, and the features from the other view help with further deblurring. The quantitative and qualitative influence of the mPAM module is shown in Table 5 and Fig. 10, respectively.

#### Effect of decoder

Since the output of the mPAM module has 32 filters, we use a 2D convolution after the mPAM to create a 3 channel output to add to the blurry input frames. We remove the convolutional decoder and add the output of the mPAM module to the blurry middle frame to create the deblurred output frames. The result is shown in the third row of Table 4, which shows the importance of the decoder module. This table shows that the decoder module includes 21.7 million of parameters, a high number compared to other parts of our model. In the future, we will work on reducing the complexity of the decoder module.

**Table 6:** Impact of the number of input frames ($N\_frames$) on the performance of the proposed model on the Stereo Blur^42^ dataset.

$N\_frames$	PSNR	SSIM
3	32.29	0.9507
5	34.06	0.9742
7	34.22	0.9777

$N\_frames=5$ demonstrates a favorable trade-off between performance and complexity.

#### Effect of consecutive frames numbers

In Sect. Quantitative results , we highlighted the use of a sequence consisting of 5 consecutive frames in our experiments. Here, we investigate how altering the number of input frames affects the performance of our model. Table 6 presents a comparative analysis across different frame counts, specifically $N\_frames = 3$, 5, and 7. The results demonstrate that selecting $N\_frames=5$ yields optimal performance for stereo video deblurring. Notably, our proposed method exhibits sub-optimal performance with $N\_frames=3$, while only marginal improvements are observed with $N\_frames=7$. Therefore, choosing $N\_frames=5$ strikes a favorable balance between performance and complexity.

### Limitations

The increased number of model parameters in the proposed technique compared to image and 2D video deblurring methods is one of its drawbacks. As shown in Table 1, our model includes 38.4 million parameters, compared to 19.9 million for UNet-Deblur^43^, 32.4 million for Pan et al.^34^, 21.02 million for Son et al.^35^, and 8.68 million for DAVANet^42^. This increase in parameter count is logical given that our proposed method addresses video deblurring with additional stereo-related information compared to 2D image-based and video-based methods. The inclusion of the temporal dimension inherently results in a model with higher complexity, such as using 3D convolutions instead of 2D. However, in the future, we aim to refine the modules of the overall architecture to make it more lightweight.

## Conclusions

This paper proposed a new model for deblurring stereoscopic videos, marking the first Transformer-based stereo video deblurring method. We design its self-attention and feed-forward layers specifically for stereoscopic video deblurring. Additionally, we develop a method for fusing stereo information to enhance deblurring further. Our approach utilizes neighboring frames of a monocular view and corresponding stereo view to deblur the target frame. Extensive experiments demonstrate that our proposed approach outperforms both image and video-based deblurring methods on two benchmark datasets. In future work, we plan to optimize different parts of the proposed model to reduce complexity. Specifically, we aim to redesign the decoder to achieve comparable performance with fewer parameters. Additionally, we intend to refine the motion compensation module to focus more on the motion or salient parts of stereo videos.

## Data Availability

The source code for this work is available upon request to corresponding author(s).
